# The circadian system in cystic fibrosis mice is regulated by histone deacetylase 6

**DOI:** 10.1152/ajpcell.00248.2022

**Published:** 2022-09-05

**Authors:** Eric Barbato, Rebecca Darrah, Thomas J. Kelley

**Affiliations:** Department of Genetics and Genome Sciences, Case Western Reserve University, Cleveland, Ohio

**Keywords:** circadian, cystic fibrosis, HDAC6, melatonin, microtubule

## Abstract

Disordered sleep experienced by people with cystic fibrosis (CF) suggest a possible disruption in circadian regulation being associated with the loss of cystic fibrosis transmembrane conductance regulator (*Cftr*) function. To test this hypothesis, circadian regulation was assessed in an F508del/F508del CF mouse model. CF mice exhibited significant alterations in both timing of locomotor activity and in mean activity per hour in both light-dark (LD) and dark-dark (DD) photoperiods compared with wild-type (WT) controls. It was also noted that in DD periodicity increased in CF mice, whereas shortening in WT mice as is expected. CF mice also exhibited altered timing of circadian gene expression and a reduction of melatonin production at all time points. Mechanistically, the role of microtubules in regulating these outcomes was explored. Mice lacking expression of tubulin polymerization promoting protein (*Tppp*) effectively mimicked CF mouse phenotypes with each measured outcome. Depleting expression of the microtubule regulatory protein histone deacetylase 6 (*Hdac6*) from CF mice (CF/*Hdac6*) resulted in the reversal of each phenotype to WT profiles. These data demonstrate an innate disruption of circadian regulation in CF mice and identify a novel microtubule-related mechanism leading to this disruption that can be targeted for therapeutic intervention.

## Graphical abstract

**Figure:**
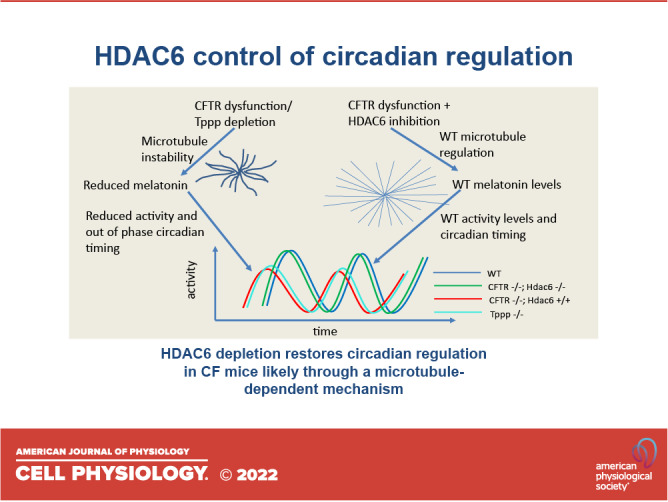

## INTRODUCTION

Sleep disturbances are a common clinical complaint of people with cystic fibrosis (CF). It has long been a clinical assumption that CF-related respiratory symptoms were the primary cause of the widespread sleep disturbances in CF (1, 2). However, children with CF experience similar nocturnal respiratory profiles to those of healthy children but still suffer from disrupted sleep (3). These data suggest there may be other factors contributing to poor sleep quality in individuals with CF. Other studies have determined that CF sleep disturbances are consistent with circadian rhythm (CR) phase delays (4, 5). We recently evaluated circadian clock gene expression profiles (*Clock*, *Bmal1*, *Period1*, *Period2*, *Cryptochrome1*, and *Cryptochrome2)* in the brain, colon, fat, jejunum, lung, and skeletal muscle of CF and non-CF mice. We found significant differences in the gene expression profiles of CF mice, with increases in *Clock* (brain and jejunum), *Bmal1* (jejunum), and *Cry2* (brain) in CF tissues (6). Given this evidence of CR gene expression changes in CF, characterizing the specific CR phenotypic alterations that occur in CF and defining the mechanisms responsible for those alterations became primary goals.

We have previously shown that CF cells exhibit specific changes to microtubule regulation consisting of reduced acetylation and slower rates of microtubule reformation indicating less stable microtubules (7, 8). These microtubule alterations have multiple consequences with regard to CF cell biology and in vivo phenotypes. At the cellular level, microtubule alterations result in an increase in retrograde transport of endosomes with endosomes accumulating in the perinuclear region, free-cholesterol accumulation in those endosomes resulting in elevated de novo cholesterol synthesis and elevated membrane cholesterol content and increased inflammatory signaling (8–11). Each of these cellular phenotypes is reversible by inhibiting the microtubule modifying protein histone deacetylase 6 (*Hdac6*; 7, 10). Knocking-out expression of *Hdac6* in a CF mouse model reveals that characteristic CF phenotypes are reversed, including the normalization of inflammatory responses and bacterial clearance rates of mice challenged with *Pseudomonas aeruginosa* airway infection (12). It was also shown that the reduced growth and depression-like behaviors characteristic of CF mouse models were reversed by *Hdac6* knockout (13, 14). These data demonstrate the *Hdac6*-dependent events control multiple phenotypes in CF cells and mouse models. A goal of this study is to determine if the microtubule pathway also contributes to CR-related gene expression and any related CR phenotypes in CF mice.

Microtubule-related regulation of CR has been shown previously. Knock-out of the microtubule-stabilizing protein stable tubulin only polypeptide (STOP; also known as MAP6) results in mice that have sleep disturbance and reduced overall activity and rhythm expression, though broad circadian mechanisms are intact (15, 16). Also, an earlier study demonstrated that microtubules were essential for melatonin receptor MT1 localization and control of circadian activity rhythms (17). We have recently demonstrated that mice lacking expression of the microtubule regulating protein tubulin polymerization promoting protein (*Tppp*) exhibit CR-related gene expression changes, reduced melatonin production, and altered circadian activity rhythms (18). *Tppp* is particularly interesting in the realm of CF studies since it regulates the two aspects of microtubule function we have found to be altered in CF, tubulin polymerization rates and tubulin acetylation through modulation of *Hdac6* activity (19). We have shown previously that knocking down *Tppp* expression recapitulates CF-like cellular phenotypes in airway epithelial cells (10). The *Tppp* locus was also identified in genome-wide association study (GWAS) as a modifier of CF airway disease severity (20).

In this study, we will test the hypothesis that CF mice exhibit CR-related alterations that are dependent on *Hdac6*-dependent mechanisms. We also compare CF CR phenotypes to those of *Tppp* knockout mice to further test whether these phenotypes are due to microtubule instability in CF. It is found that CF mice exhibit reduced activity, altered circadian regulation, and reduced melatonin production that are normalized to wild-type (WT) levels by depletion of *Hdac6* expression. A model of microtubule dysfunction, *Tppp*^−/−^ mice, mimic CF phenotypes at every level providing further evidence that microtubule regulation is altered in CF and impacts clinically relevant phenotypes.

## MATERIALS AND METHODS

### Categories for Evaluation

To test our hypotheses, we compared four groups of C57Bl6 mice: wildtype (WT), CF (*Cftr* F508del/F508del), *Tppp*^−/−^, and CF/*Hdac6* (*Cftr* F508del/F508del, *Hdac6*^−/−^). These mice were compared in three categories. The first category was locomotor activity, which was measured continuously in identical environments for a total of 4 wk; 2 wk in 12:12 light-dark (LD) conditions followed by 2 wk in dark-dark (DD) conditions. The second category was clock gene expression, which included serial quantification of *Clock*, *Bmal1*, *Period1*, *Period2*, *Cryptochrome1*, and *Cryptochrome2* mRNA isolated from suprachiasmatic nucleus (SCN) tissue. The third and final category was serial serum melatonin concentration, measured by ELISA. Both clock gene expression and serum melatonin were measured at four time points throughout one 24-h LD cycle.

### Animals and Measurement of Locomotor Activity

The experimental protocols described in this report including animal breeding, housing, surgery, behavioral testing, euthanize, and tissue collection, were approved by the Institutional Animal Care and Use Committee of Case Western Reserve University, Cleveland, Ohio. C57Bl/6 mice of four genotype groups were utilized for these experiments as listed earlier. Each group consisted of eight mice: four males and four females. Mice in both groups ranged from 7 to 14 wk old, averaging 10.4 wk at the start of the data collection period. Each mouse was implanted with a subcutaneous telemetry device and allowed a 5-day recovery period before acclimation to a light-tight circadian cabinet in which the mice were housed for the duration of the experiments with access to food and water ad-libitum.

Different mice (of the same sexes, ages, and genotypes) were used for the gene expression and melatonin experiments. Our intention was to minimize variables that might contribute to variation in these measurements (i.e., having undergone surgical device implantation and having been exposed to different photoperiods). For the gene expression and melatonin experiments, eight mice of each genotype were placed in the cabinet in 12:12 LD photoperiod. Over the course of 1 day, mice were taken from the cabinet in groups of eight (two of each genotype) for euthanasia at four time points: ZT 6, ZT 12, ZT 18, and ZT 0.

Implantation surgery was performed in sterile conditions under anesthesia with nebulized isoflurane. Once a surgical plane of anesthesia was confirmed by toe pinch, each mouse was pretreated with carprofen for analgesia, secured to the heated surgical table, and a ∼2 sq cm area was shaved on the dorsal fur between the base of the skull and scapulae. Following preparation of the area with betadine, a ∼1 cm superficial incision was made and gently spread posteriorly with surgical scissors. Using forceps, a G2 E-Mitter telemetry device (Starr Life Sciences), which had previously been sterilized and rinsed in sterile saline, was implanted in the incision site. The surgical incision was then closed using a single wound clip, and the mouse was placed into a standard mouse cage atop a heating pad. Postsurgical analgesia was administered as needed for the following 3 days of recovery.

After the 5-day recovery period, mice were placed one per cage atop an ER-4000 Energizer/Receiver (Starr Life Sciences), a receiving plate slightly larger than a standard mouse cage designed to receive telemetry data from the E-Mitter device. Each cage/receiver combination was placed in a custom circadian cabinet (Actimetrics), which is designed to house eight mouse cages simultaneously and is shielded from exterior light. The mice were then allowed to acclimate to the cabinet environment for 1 wk at constant temperature (22°C ± 3°C) on a constant 12:12 LD photoperiod identical in timing to the lights of the room in the animal facility in which they had been previously housed. Following the acclimation period, the E-Mitter/ER-4000 equipment was activated and continuous gross motor activity data were collected for a period of 2 wk (14 days). For the DD experiments, data collection was stopped, the cabinet lights were turned off, and the mice were allowed a 2-wk acclimation period before the DD data collection period began. Motor activity data were then collected for an additional 2 wk (14 days) under these conditions.

Locomotor activity data were collected using VitalView 5.1 (Starr Life Sciences) and analyzed using Clocklabs Analysis 6.0 (Actimetrics). Double-plotted actograms were generated using Clocklabs Analysis.

### Tissue Collection, RNA Extraction, and Gene Expression Analyses

On the day of euthanasia, animals were taken from the circadian cabinet in groups of eight (two of each genotype) and euthanized at ZT 6, ZT 12, ZT 18, and ZT 0. Mice were euthanized via CO_2_ exposure, and SCN was immediately dissected and flash frozen in liquid nitrogen. Each frozen tissue sample was then stored at −80°C for later use. Total RNA was extracted from each sample using the RNeasy Mini Kit (Qiagen) according to the manufacturer’s protocol. RNA quality and concentration were quantified using a Nanodrop spectrophotometer (Thermo Fisher), and 1 µg of total RNA was converted to cDNA using the qScript cDNA synthesis kit (Quantabio) according to the manufacturer’s protocol.

To determine the relative expression of each gene transcript, we performed quantitative real-time PCR using standard TaqMan assays (Thermo Fisher) with β-actin as an endogenous control. mRNA transcripts evaluated for expression were: *Clock* (Mm00455950_m1), *Bmal1* (Mm00500226_m1), *Period1* (Mm00501813_m1), *Period2* (Mm00478113_m1), *Cryptochrome1* (Mm00514392_m1), and *Cryptochrome2* (Mm01331539_m1). Each reaction was denatured at 95°C and amplified at 60°C for 40 cycles alongside cDNA controls with no reverse-transcriptase to verify the absence of detectable genomic carryover in the RNA isolates. Cycle thresholds (Cts) were calculated using QuantStudio software (Thermo Fisher), and mean Ct difference (ΔCt) was calculated between each gene of interest and endogenous controls. Genotype groups (CF vs. WT, *Tppp*^−/−^ vs. WT, and CF/*Hdac6* vs. WT) were then compared by calculating ΔCt difference between each group for each transcript (ΔΔCt). Fold differences of the experimental groups (2^−ΔΔCt^) are reported as a fold change compared with the WT control group.

### Serum Extraction and Melatonin Measurement

It has been reported that rodents produce very small concentrations of melatonin, which are often difficult to detect (21). Consequently, to accurately evaluate circulating melatonin concentration at multiple time points, it was necessary to use as much serum as possible for each sample, prohibiting a survival extraction technique such as orbital or tail vein blood draw. Immediately following euthanasia and alongside tissue dissection, a cardiac puncture was performed, and whole blood was collected via a sterile bulb pipette. Whole blood was placed in sterile 1.5 mL serum separator tubes (BD) and spun at 10,000 *g* for 7 min at room temperature to separate serum from other blood components. Serum was then extracted, lyophilized using a SpeedVac at room temperature, and frozen at −20°C for later use. Lyophilized serum was reconstituted and melatonin concentration was measured according to the manufacturer’s protocol using a Melatonin ELISA Kit (ENZ-KIT150-0001; Enzo Life Sciences).

### Ussing Chamber Analysis

Short-circuit current measurements were performed in *Tppp*^−/−^ mouse nasal epithelial (MNE) cells. Primary MNE tissue was excised from *Tppp*^−/−^ mice. They were grown at 37°C in a 95% O_2_-5% CO_2_ incubator on T-75 flasks (Corning Inc., Corning, NY) in F12 medium containing Y-27632, penicillin, and streptomycin. Cells were obtained from the Case Western Reserve University (CWRU) CF center cell culture core facility. After 5 wk, the establishment of electrically tight cultures was determined by Ussing chamber measurements at which time electrophysiologic analysis was completed. The epithelial monolayers were bathed with symmetrical Krebs bicarbonate ringer’s solution and maintained under short-circuit conditions. Amiloride (100 µM), forskolin (10 µM), and *CFTR*^inh172^ (10 µM) were added sequentially to inhibit Na^+^ absorption, stimulate *CFTR*-dependent Cl^–^ secretion, and inhibit *CFTR* activity to determine how much current was due to *CFTR* function, respectively.

### Statistics

#### Locomotor activity.

One-way ANOVAs were conducted to compare relevant phenotypic characteristics of the experimental groups to the WT group for which statistical significance was considered *P* < 0.05, and multiple comparisons were accounted for by using Tukey’s honest significant difference (HSD) test.

#### Gene expression.

Repeated measures ANOVAs were conducted to compare average fold difference between the experimental groups and the WT group for each transcript at each time point. After conducting the Bonferroni correction for multiple comparisons, statistical significance for these comparisons was considered *P* < 0.008.

#### Melatonin measurements.

One-way ANOVAs were conducted to compare mean serum melatonin concentration between the experimental groups and the WT group at each time point. After conducting the Bonferroni correction for multiple comparisons, statistical significance for these comparisons was considered *P* < 0.01.

### Study Approval

All animal experiments were approved by the Research Animal Resource Center of Case Western Reserve University (IACUC 2021-0071: elucidating metabolic phenotypes using CF mouse models).

## RESULTS

### Locomotor Activity

**Figure 1.**
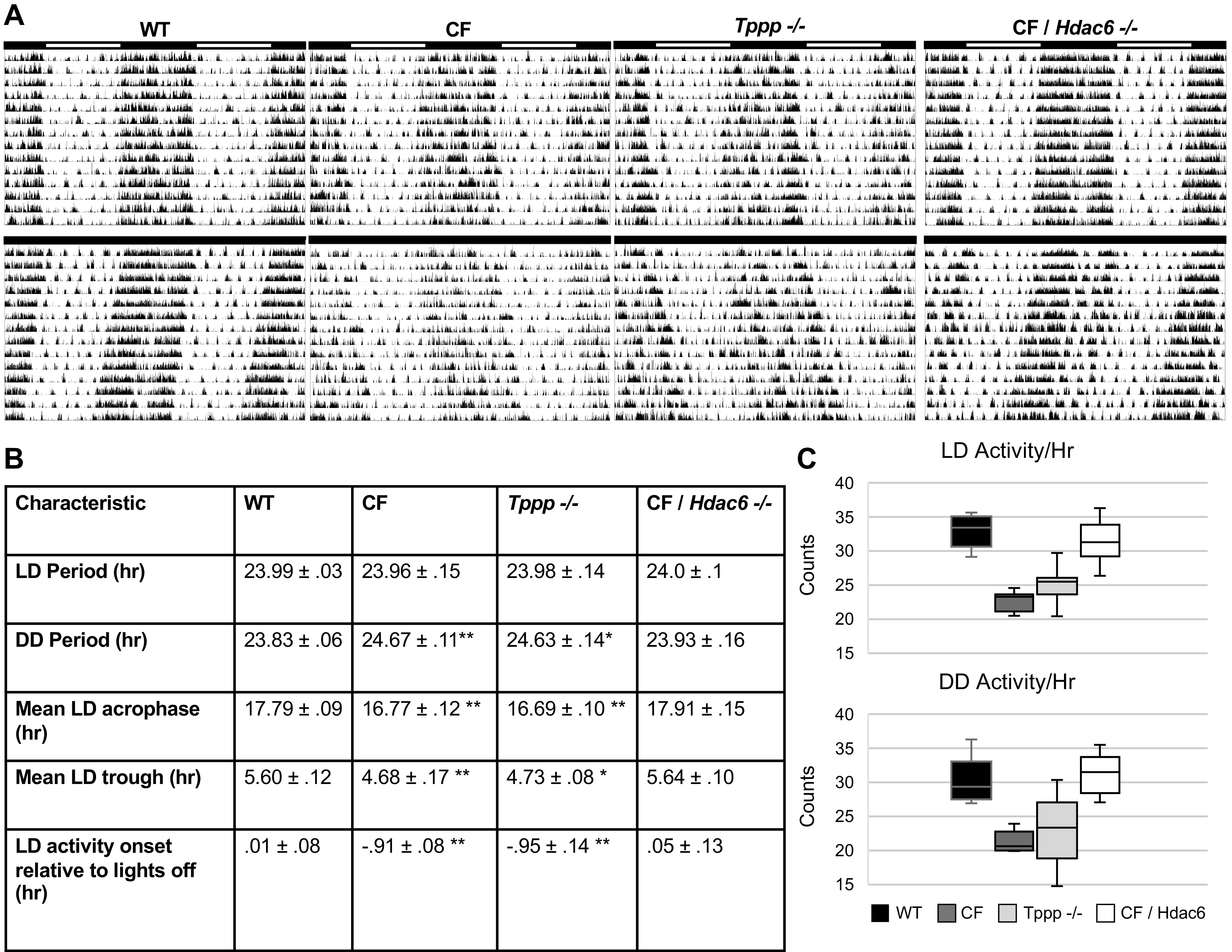
Targeted deletion of *Hdac6* restores circadian phenotypes in cystic fibrosis mice. *A*: representative actograms of WT, CF, *Tppp^−/−^*, and CF/*Hdac6^−/−^* mice during 12/12 LD photoperiod (*top*) and DD photoperiod (*bottom*). Actogram data are double plotted (48 h/line) with light schedule indicated by the black and white bars at the top of each actogram (white represents lights on, black representing lights off). *B*: characteristics of locomotor activity in WT (*n* = 8), CF (*n* = 8), *Tppp^−/−^* (*n* = 8), and CF/*Hdac6^−/−^* (*n* = 8) mice during 12/12 LD photoperiod and DD photoperiod. Values are displayed as means ± SE. *Statistical significance at the 0.05 level compared with the WT value. **Significance at the 0.01 level compared with the WT value. *C*: mean activity per hour measured in counts in WT (*n* = 8), CF (*n* = 8), *Tppp^−/−^* (*n* = 8), and CF/*Hdac6^−/−^* (*n* = 8) mice during 12/12 LD photoperiod and DD photoperiod. Values are displayed as means ± SE. *Statistical significance at the 0.05 level. CF, cystic fibrosis; DD, dark-dark; *Hdac6*, histone deacetylase 6; LD, light-dark; *Tppp,* tubulin polymerization promoting protein; WT, wild type.

It is hypothesized in this study that there are alterations in circadian regulation in CF that are due to disruptions in microtubule stability. To test this hypothesis, we used four mouse models: a WT control, a F508del CF mouse model, *Tppp*^−/−^ mice to model microtubule instability, and a CF model crossed with an *Hdac6*^−/−^ mouse line as a means to correct the microtubule issues in CF (CF/*Hdac6*). In these mouse models, locomotor activity was assessed in both 12 h light/12 h dark (LD) and dark-dark (DD) photoperiods to begin assessing circadian regulation. CF and *Tppp*^−/−^ mice showed numerous differences in locomotor activity characteristics throughout both photoperiods when compared with WT. Locomotor activity in CF/*Hdac6* mice was not different from WT mice, but significantly elevated when compared with CF and *Tppp*^−/−^ mice (Fig. 1*A*). Locomotor activity characteristics of all four groups can be found in Fig. 1*B.*

In LD photoperiod conditions, periodicity (LD period) remained unchanged across all four groups. DD periodicity, however, was significantly longer in the CF (24.67 h) and *Tppp*^−/−^ (24.63 h) groups compared with WT controls (23.83 h). Mean LD acrophase occurred significantly earlier in the CF (16.77 h) and *Tppp*^−/−^ (16.69 h) groups when compared with WT controls (17.79 h). Similarly, mean LD trough of the CF (4.68 h) and *Tppp*^−/−^ (4.73 h) groups were significantly earlier than WT (5.6 h). In addition, mean activity onset relative to lights off was earlier in the CF (−0.91 h) and *Tppp*^−/−^ (−0.95 h) groups compared with WT (0.01 h). Notably, all four parameters which were altered in the CF and *Tppp*^−/−^ groups (DD period, LD acrophase, LD trough, and LD activity onset) were corrected to near-WT values in the CF/*Hdac6* group.

In addition to the differences, we observed in timing of locomotor activity characteristics, we also noted significant differences in mean activity per hour in both LD and DD photoperiods (Fig. 1*C*). One-way ANOVA revealed a significant difference in LD activity per hour between at least two groups [*F*_LD_(3, 28) = (31.13), *P* = < 0.001]. Tukey’s honest significant difference test for multiple comparisons found that mean LD activity per hour was different between CF and WT (*P* = < 0.001, 95% C.I. [−10.25, 1.24]) and *Tppp*^−/−^ and WT (*P* = < 0.001, 95% C.I. [−7.76, 1.24]). An additional one-way ANOVA revealed a similar difference in mean DD activity per hour in at least two groups [*F*_DD_(3, 28) = (17.42), *P* = < 0.001]. Again, Tukey’s HSD test for multiple comparisons found that mean DD activity per hour was different between CF and WT (*P* = < 0.001, 95% C.I. [−8.99, 1.71]) and *Tppp*^−/−^ and WT (*P* = 0.001, 95% C.I. [−7.18, 1.24]). No statistically significant differences were found between the WT and CF/*Hdac6* groups for either parameter.

These differences in timing and amplitude of locomotor activity characteristics can be observed qualitatively in the representative LD and DD actograms (Fig. 1*A*). In these double-plotted actograms, locomotor activity in LD photoperiod (*top row*) is largely unchanged across the groups, though onsets and offsets are clearly earlier, and total activity is clearly reduced in the CF and *Tppp*^−/−^ groups when compared with WT and CF/*Hdac6*. When placed in DD photoperiod (*bottom row*), the CF and *Tppp*^−/−^ mice lose some stability of their rest-activity rhythms, indicated by the worsening sporadic activity signals during their inactive phases. In addition, the WT and CF/*Hdac6* mice conform to typical murine behavior in DD photoperiod, gradually shortening their periodicity over time as indicated by the gradual shift to the left. However, under identical conditions, the CF and *Tppp*^−/−^ groups slowly increased in periodicity, as indicated by the gradual shift to the right.

These data demonstrate that compared with WT controls there are clear circadian changes inherent to CF mice. Mechanistically, the functional changes in circadian regulation in CF mice are mimicked by the *Tppp*^−/−^ mice that model microtubule instability. Circadian regulation is also corrected in CF mice by the depletion of the *Hdac6* expression, further supporting the hypothesis that microtubule dysregulation in CF is driving these changes.

### Gene Expression

**Figure 2.**
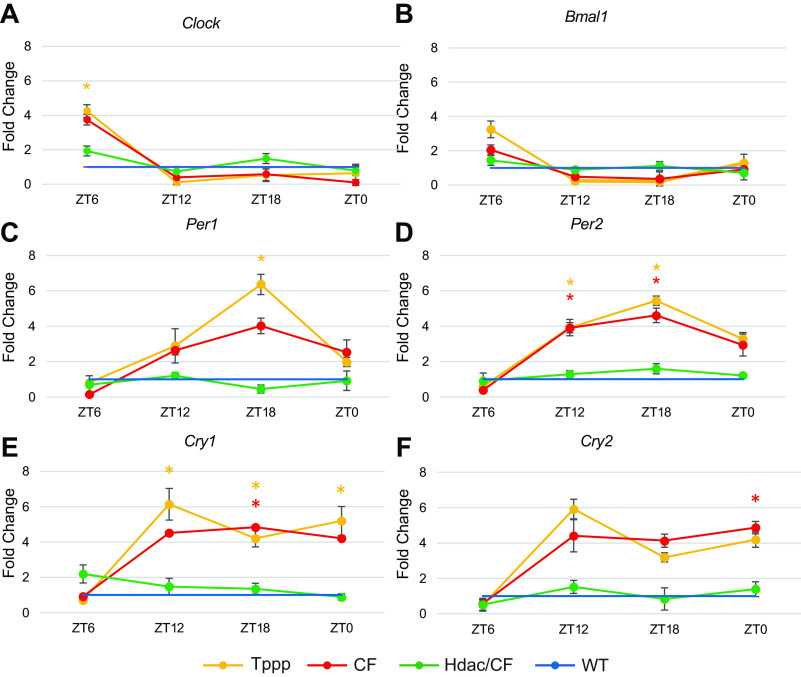
Deletion of *Tppp* causes clock gene expression changes that mimic CF, which are reverted to WT patterns by deletion of *Hdac6*. Serial measurements of *Clock* (*A*), *Bmal1* (*B*), *Per1* (*C*), *Per2* (*D*), *Cry1* (*E*), *Cry2* (*F*) in SCN tissue of CF (red, *n* = 8), *Tppp*^−/−^ (yellow, *n* = 8), and CF/Hdac6 (green, *n* = 8) animals. All measurements are compared with WT (blue, *n* = 8) at four time points: ZT 6, ZT 12, ZT 18, and ZT 0. All values are displayed as fold change of the experimental group relative to WT (the WT value for each transcript is represented by 1.00 on the *y*-axis). Yellow * represent statistical significance of the *Tppp*^−/−^ group compared with WT. Red * represent statistical significance of the CF group compared with WT. Error bars represent means ± SE. CF, cystic fibrosis; *Hdac6*, histone deacetylase 6; *Tppp,* tubulin polymerization promoting protein; WT, wild type.

Gene expression was also examined in these mouse models to further investigate the level of regulatory changes in the circadian system. Significant differences were detected in the expression of all six measured transcripts at one or more timepoints in the CF and *Tppp*^−/−^ groups when compared with the WT group (Fig. 2).

At ZT 6, *Clock* was significantly overexpressed in the *Tppp*^−/−^ group compared with WT (*P* = 0.005) and trended up in the CF group, though was not significant after being adjusted for multiple comparisons (*P* = 0.009). One-way ANOVA did not reveal any other differences in mean expression of *Clock* between the four groups that were significant at the adjusted level (*P* = 0.008).

At ZT 12 and ZT 18, expression of *Bmal1* trended down in the CF and *Tppp*^−/−^ groups, though was not significantly different than WT at any timepoint once adjusted for multiple comparisons.

*Per1* trended up at ZT 12, ZT 18, and ZT 0 in both the CF and *Tppp*^−/−^ groups when compared with WT. At ZT 18 only, *Per1* was significantly overexpressed in the *Tppp*^−/−^ group compared with WT. Despite their upward trend, no other comparisons of *Per1* expression were statistically significant.

At ZT 12 and ZT 18, *Per2* was significantly overexpressed in both the CF group (*P* = 0.007, *P* = 0.003) and *Tppp*^−/−^ group (*P* = 0.008, *P* = 0.001) when compared with WT. Though both groups again trended up at ZT 0, neither comparison was statistically significant compared with WT at that timepoint.

*Cry1* expression was significantly upregulated in the *Tppp*^−/−^ group compared with WT controls at ZT 12 (*P* = 0.007), ZT 18 (*P* = 0.005), and ZT 0 (*P* = 0.007). Expression of *Cry1* also trended up in the CF group at all three of those timepoints but was only significantly different from the WT group at ZT 18 (*P* = 0.005). Finally, *Cry2* was significantly overexpressed in the CF group at ZT 0 (*P* = 0.005), and trended up in the CF and *Tppp*^−/−^ groups at ZT 12, ZT 18, and ZT 0. No significant differences in *Cry1* or *Cry2* expression were detected by one-way ANOVA at ZT 6.

Notably, no trends or significant differences were observed between the CF and *Tppp*^−/−^ groups or the CF/*Hdac6* group and WT controls in any measured transcript at any timepoint. Ct values of the endogenous control were stable throughout the experiment, ranging from 17.19 to 19.59.

### Melatonin Concentration

**Figure 3.**
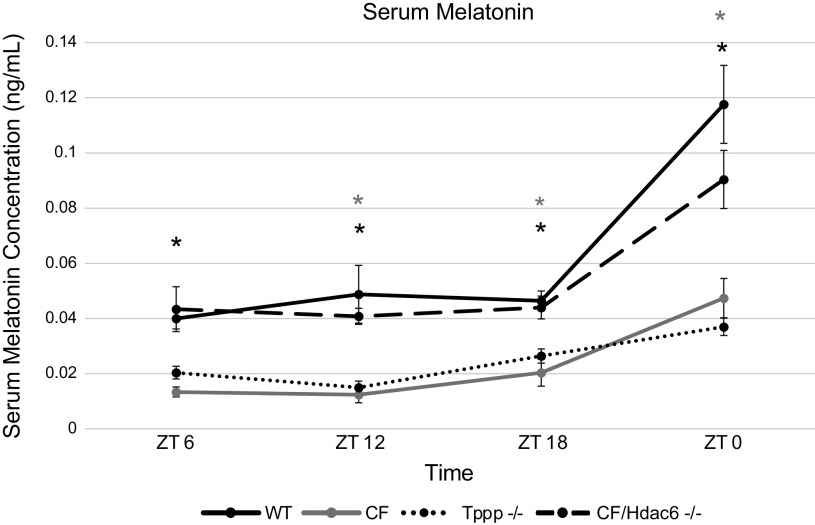
Microtubule dysfunction and CF independently result in decreased serum melatonin, which is corrected in CF by restoration of microtubule function. Serum melatonin concentration; WT (black) vs. CF (gray) vs. *Tppp*^−/−^ (dotted) vs. CF/*Hdac6* (dashed) at four timepoints (*n* = 3/group/timepoint). Mean concentrations are displayed in nanograms per milliliter of serum. Black * represent statistical significance of the *Tppp*^−/−^ group compared with WT. Gray * represent statistical significance of the CF group compared with WT. Error bars represent means ± SE. CF, cystic fibrosis; *Hdac6*, histone deacetylase 6; *Tppp,* tubulin polymerization promoting protein; WT, wild type.

Though not the only regulator, melatonin is a key regulator of the circadian system and its production over time was examined in the mouse models. Significant differences were detected in serum melatonin concentration between the *Tppp*^−/−^ and WT groups at all four timepoints. In addition, the rate at which serum melatonin concentration increased across the four measurement points was dampened in the *Tppp*^−/−^ group compared with the control group (Fig. 3).

At the first timepoint (ZT 6), mean melatonin concentration was significantly lower in the CF group (0.014 ng/mL) compared with WT control (*P* = 0.005). At ZT 12, mean serum melatonin concentration in both the CF group (*P* = 0.002) and *Tppp*^−/−^ group (*P* = 0.004) were significantly lower than the WT control group. At the third timepoint (ZT 18), mean concentration of the CF group (*P* = 0.01) and the *Tppp*^−/−^ group (*P* = 0.011) were again lower than WT controls. Finally, at ZT 0, mean concentrations of the CF group (*P* < 0.001) and the *Tppp*^−/−^ group (*P* < 0.001) were once more significantly lower than the WT, showing the largest difference in group means at this timepoint.

### *CFTR* Expression and Function

**Figure 4.**
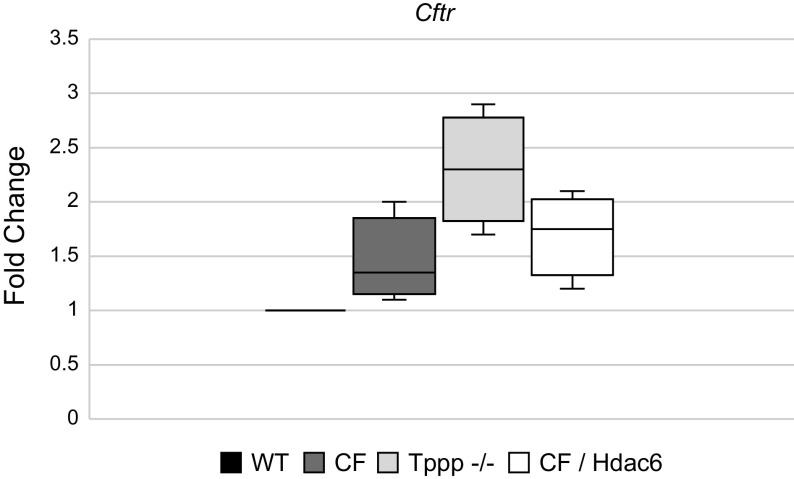
Cystic fibrosis transmembrane conductance regulator *(Cftr*) mRNA is expressed in suprachiasmatic nucleus tissue. Box and whisker plot showing fold change of *Cftr* expression in CF (*n* = 4), *Tppp*^−/−^ (*n* = 4), and CF*/Hdac6*^−/−^ (*n* = 4) animals relative to WT (*n* = 4). All values are displayed as fold change of the experimental group relative to WT, which is represented as 1.00 on the *y*-axis. CF, cystic fibrosis; *Hdac6*, histone deacetylase 6; *Tppp,* tubulin polymerization promoting protein; WT, wild type.

**Figure 5.**
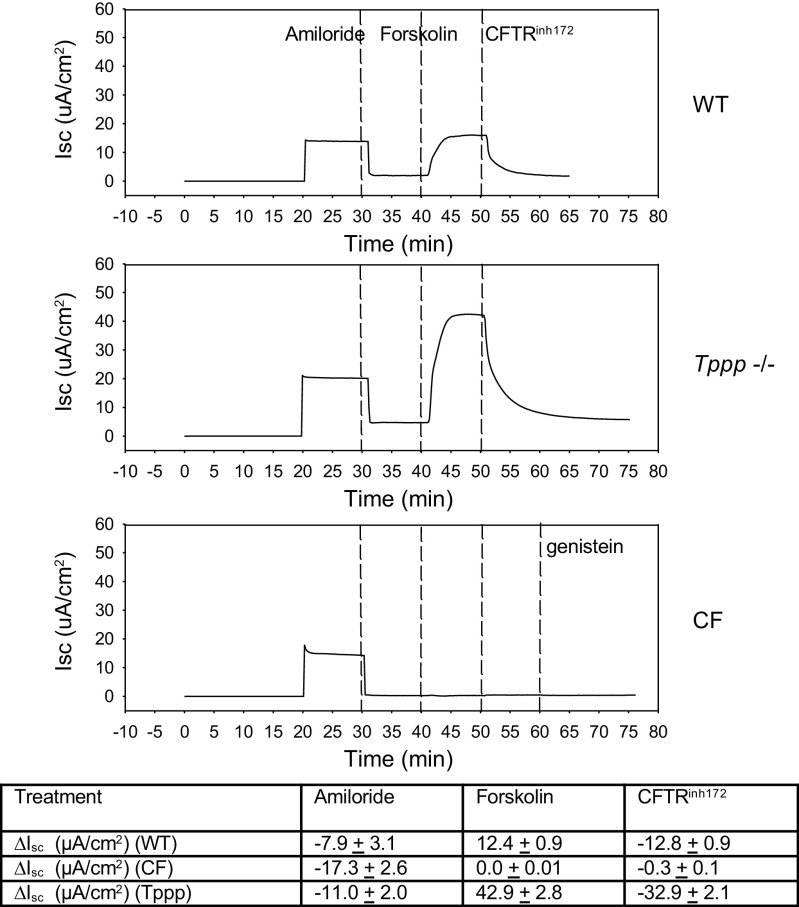
CFTR function in WT, *Tppp*^−/−^, and F508del (CF) mouse nasal epithelial (MNE) cells. Representative trace of short-circuit current (*I*_sc_/cm^2^) in MNE treated with amiloride (100 µM), forskolin (10 µM), and *CFTR*^inh172^ (10 µM) as indicated by dotted lines. CF mice were also treated with genistein (30 µM). Changes in *I*_sc_ (DIsc/cm^2^) induced by each compound. Data represent *n* = 3–6 replicates with means ± SE shown. CF, cystic fibrosis; *Tppp,* tubulin polymerization promoting protein; WT, wild type.

Aforementioned data demonstrate that *Tppp*^−/−^ mice effectively replicate CF phenotypes with regard to melatonin production and circadian regulation. To ensure that these phenotypes are not due to an adverse effect of *Tppp* depletion on *CFTR* expression or function, we examined CFTR expression in suprachiasmatic nucleus and surrounding tissue from each mouse model as well as CFTR function in primary mouse nasal epithelial cells (MNE) from WT, F508del (CF), and *Tppp*^−/−^ mice. MNE were cultured and subjected to Ussing chamber analysis to determine relative CFTR function. Expression data demonstrate *Cftr* trasnscript levels in the SCN of each mouse model relative to WT content. Of note, *Tppp*^−/−^ exhibits a more than twofold increase in *Cftr* expression (Fig. 4). Functional analysis of CFTR activity in MNE reveals a similar finding. As shown in Fig. 5, after amiloride inhibition of sodium transport, forskolin stimulates significant current that is sensitive to the CFTR-selective inhibitor *CFTR*^inh172^. Similarly, we have previously demonstrated that correction of CF phenotypes in CF/*Hdac6* mice is not due to any restoration of CFTR function (13). These data demonstrate that *Tppp*^−/−^ mice have elevated *Cftr* expression in the SCN and elevated *CFTR* function compared with WT mice. It can be concluded that CF-like phenotypes in *Tppp*^−/−^ mice are not the result of adverse secondary impacts on CFTR. The relationship between *Tppp* depletion and elevated *Cftr* expression and function needs to be further explored.

## DISCUSSION

The goals of this study were to determine if CF mice exhibit inherent alterations to CR regulation that could account for the patient clinical manifestation of disrupted sleep, and if there is CR dysregulation, also determine the mechanism of those CF-related changes. The first significant finding of this study is that CF mice do exhibit clear changes in CR regulation encompassing sporadic and reduced overall locomotor activity, phase shift in activity onset, altered timing of CR-related gene expression, and reduced melatonin expression. The second finding of the study details that the CR phenotypes in CF mice are directly related to microtubule changes previously shown to be characteristic of CF cells. CF-related CR phenotypes are replicated by the mouse model of microtubule instability, *Tppp*^−/−^ mice, and reversed to WT profiles by the depletion of the tubulin deacetylase *Hdac6* expression.

Sleep disturbances in people with CF are typically attributed to severe lung disease and chronic cough (22). However, pediatric patients that have yet to develop severe lung disease also report inability to sleep, suggesting more innate mechanisms are leading to this phenotype. The significance of these studies is that the presence of circadian system disruptions in CF mice strongly suggest that the absence of *CFTR* function alone is sufficient to cause these changes. Though there are reports of lung structure and function changes in CF mice (23), they do not exhibit airway obstruction due to excessive mucus production (24). CF mice clearly exhibit a phase shift in circadian response that is reflected both in activity profiles and CR regulatory gene expression timing. These data in mice are consistent with reports demonstrating circadian phase changes in patients with CF (4, 5). The presence of inherent changes to circadian regulation in response to the reduced function or absence of *CFTR* is significant but does not clarify the mechanisms linking *CFTR* function to the circadian system.

To address a potential mechanism linking *CFTR* to CR regulation, we explored the potential role of the microtubule alterations we have previously found in CF cells. Microtubules in CF exhibit at least two distinct alterations compared with non-CF cells: reduced acetylation and slower rates of reformation (7, 8). These microtubule changes have been shown to impact several cellular phenotypes including intracellular distribution of cholesterol, endosomal trafficking, and inflammatory signaling, and each of these cellular phenotypes is reversible by inhibition of histone deacetylase 6 (*Hdac6*), a predominantly cytosolic deacetylase that deacetylates tubulin and other targets (7). The most notable target, at least indirectly, is CFTR itself. One concern is that *Hdac6* depletion is reversing CF phenotypes through increasing CFTR function in our mouse model. Researchers have demonstrated that Hdac6 interacts directly with ubiquitinated CFTR and participates in its trafficking to aggresomes (25, 26). Similarly, Hutt et al. (27) have reported that pan-Hdac inhibition can increase F508del CFTR processing due to interference with autophagy, resulting in more F508del CFTR being localized to the plasma membrane, though an emphasis was placed on *Hdac7* in this manuscript. *Hdac6* also regulates the acetylation of the chaperone Hsp90 whose function can influence not only CFTR trafficking but multiple pathways including microtubule stability (28, 29). Though we cannot rule out every pathway potentially impacted by *Hdac6* depletion on CF phenotypes, we have examined the impact of Hdac6 depletion on CFTR function in this model previously to rule out CFTR correction as a mechanism to relieve CF phenotypes. There is no impact on F508del CFTR function by *Hdac6* depletion in the mouse model used in this study (13). However, there is a significant effect of *Hdac6* depletion on CF phenotypes in vivo. Knocking out *Hdac6* expression from CF mice improves growth, reverses depression-like behavior, and normalizes inflammatory responses to bacterial infection (12–14). It has also been shown that knocking-down expression of *Tppp* in epithelial cells recapitulates CF cellular phenotypes, a significant finding since *Tppp* has been identified as a modifier of CF airway disease severity (20). As noted above in the INTRODUCTION, *Tppp* regulates the two microtubule regulatory steps identified as being altered in CF cells, microtubule acetylation and tubulin polymerization.

We have shown that mice lacking expression of *Tppp* exhibit changes in CR regulation, directly implicating microtubule stability in CR control (18). In this study, it is shown that depletion of *Hdac6* expression in CF mice completely reverses every aspect of CR dysregulation identified in CF mice, including overall activity, circadian phase regulation, gene expression timing, and melatonin expression. Based on previous findings, it is postulated that correction of microtubule function is the mechanism behind the efficacy of *Hdac6* depletion. To further test this hypothesis, CF mice were compared with *Tppp*^−/−^ mice to determine if there is consistency between CF phenotypes and those elicited by a direct manipulation of microtubule control. CF and *Tppp*^−/−^ mice prove to be indistinguishable at nearly every measure. Taken together, these data strongly indicate that microtubule disruption in CF is impacting CR control and related phenotypes.

Though *Hdac6* control of microtubule function in CF is a key mechanism in CF-related CR control, there remain questions as to why microtubule changes would impact CR outcomes. One potential mechanism would be the effect of microtubules on melatonin receptor localization. The Witt-Enderby group has demonstrated that a clear relationship between microtubule function and melatonin receptor localization. Destabilization of microtubules resulted in cytoplasmic localization of the receptor and an increase in melatonin-induced protein kinase C activation (30, 31). Functionally, the same group identified disruption of microtubules in the SCN resulted in enhanced ability of melatonin to regulate phase shifts in a rat model (17). Most directly, a study examining the impact of knocking-out the microtubule-stabilizing protein stable tubulin only polypeptide (STOP/Map6) results in mice that have sleep disturbance and reduced overall activity and rhythm expression, very similar to what is observed in CF and *Tppp*^−/−^ mice in this study (15, 16). Studies examining melatonin receptor expression are underway, but there is another mechanism that might be more directly regulating CR in these model systems; clear reduction in melatonin production in both CF and *Tppp*^−/−^ mice. Both CF and *Tppp*^−/−^ mice exhibit reduced melatonin production that is restored by Hdac6 depletion. Reduced melatonin production could certainly lead to alterations in CR gene regulation and downstream phenotypes. One study reports that patients with CF report better sleep efficiency after treatment with 3 mg melatonin for 21 days (32). However, it is not clear if reduced melatonin production is a direct effect of microtubule instability or a secondary manifestation. Another process that could impact CR control in CF is energy regulation. One of the clear findings from this study is that activity levels are reduced in both CF and *Tppp*^−/−^ mouse models. It is possible that impaired energy production due to microtubule instability results in changes to CR control as a result of reduced activity in the mice. Mitochondrial dysfunction has been reported to be characteristic of CF cells, and *Hdac6* inhibition is known to improve mitochondrial transport and function in different disease model systems (33–38). Finally, the relationship between circadian regulation and inflammation needs to be determined. Barbato et al. (6) first determined altered circadian gene regulation in brain, colon, fat, jejunum, lung, and skeletal muscle tissues. Though there is no overt chronic lung inflammation in the CF mouse model, there is evidence of chronic inflammation in the CF mouse gut (39). The Darrah group has also recently reported elevated cytokine levels in the serum of uninfected CF mice (40). Whether the presence of chronic inflammation in CF is altering CR regulation or if the lack of melatonin production and CR phase shifts in CF are impacting inflammatory responses needs to be addressed in future studies.

In summary, this study demonstrates that CR regulation is clearly different in CF mice compared with WT controls in a manner consistent with disrupted sleep phenotypes reported in patients with CF. Mechanistically, it is shown that depletion of *Hdac6* expression from CF mice completely restores CR control in all aspects measured and that *Tppp*^−/−^ mice recapitulate the CF phenotypes. It can be concluded that there are inherent CR changes in response to reduced or absent *CFTR* function. Also, *Hdac6*-sensitive mechanisms likely related to microtubule regulation have proven to be a key regulator of multiple CF phenotypes and likely a key therapeutic target.

## GRANTS

This work is supported by Cystic Fibrosis Foundation (CFF) Grant KELLEY21P0, a CFF fellowship award BARBAT20F0 (to E.B.), CFF RDP R447-CR11, and National Institutes of Health (NIH) Grant 1R01HL156928 (to T.J.K. and R.D). Mouse studies supported by the CF Mouse Resource Center at Case Western Reserve University (CFF HODGES19R1).

## DISCLOSURES

No conflicts of interest, financial or otherwise, are declared by the authors.

## AUTHOR CONTRIBUTIONS

R.D. and T.J.K. conceived and designed research; E.B. performed experiments; E.B. and T.J.K. analyzed data; E.B., R.D., and T.J.K. interpreted results of experiments; E.B. prepared figures; E.B. and T.J.K. drafted manuscript; E.B., R.D., and T.J.K. edited and revised manuscript; E.B., R.D., and T.J.K. approved final version of manuscript.
